# Inducible *Fgf13* ablation alleviates cardiac fibrosis via regulation of microtubule stability: *Fgf13* knockout attenuates cardiac fibrosis

**DOI:** 10.3724/abbs.2024075

**Published:** 2024-05-30

**Authors:** Cong Wang, Xiangchong Wang, Yiyi Zhang, Yuan Mi, Yanxue Han, Yaxin Zhi, Ran Zhao, Nanqi Cui, Qianli Ma, Huaxing Zhang, Dazhong Xue, Ruoyang Qiao, Jiabing Han, Yulou Yu, Jiaxuan Li, Mohammed Shaiea, Demin Liu, Guoqiang Gu, Chuan Wang

**Affiliations:** 1 Department of Pharmacology the Key Laboratory of Neural and Vascular Biology Ministry of Education the Key Laboratory of New Drug Pharmacology and Toxicology the Hebei Collaboration Innovation Center for Mechanism Diagnosis and Treatment of Neurological and Psychiatric Disease Hebei Medical University Shijiazhuang 050017 China; 2 Department of Pharmacology Hebei International Cooperation Center for Ion Channel Function and Innovative Traditional Chinese Medicine Hebei Higher Education Institute Applied Technology Research Center on TCM Formula Preparation Hebei University of Chinese Medicine Shijiazhuang 050091 China; 3 Department of Emergency the Fourth Hospital of Hebei Medical University Shijiazhuang 050011 China; 4 Department of Cardiology the Second Hospital of Hebei Medical University Shijiazhuang 050000 China; 5 Department of Vascular Surgery the Second Hospital of Hebei Medical University Shijiazhuang 050000 China; 6Department of Cardiac Surgery the Second Hospital of Hebei Medical University Shijiazhuang 050000 China; 7 Core Facilities and Centers Hebei Medical University Shijiazhuang 050017 China; 8 College of Basic Medicine Hebei Medical University Shijiazhuang 050017 China; 9Graduate School Hebei Medical University Shijiazhuang 050017 China

**Keywords:** FGF13, cardiac fibrosis, fibroblasts, microtubule, ROCK

## Abstract

Fibroblast growth factor (FGF) isoform 13, a distinct type of FGF, boasts significant potential for therapeutic intervention in cardiovascular dysfunctions. However, its impact on regulating fibrosis remains unexplored. This study aims to elucidate the role and mechanism of FGF13 on cardiac fibrosis. Here, we show that following transverse aortic constriction (TAC) surgery, interstitial fibrosis and collagen content increase in mice, along with reduced ejection fraction and fractional shortening, augmented heart mass. However, following *Fgf13* deletion, interstitial fibrosis is decreased, ejection fraction and fractional shortening are increased, and heart mass is decreased, compared with those in the TAC group. Mechanistically, incubation of cardiac fibroblasts with transforming growth factor β (TGFβ) increases the expressions of types I and III collagen proteins, as well as α-smooth muscle actin (α-SMA) proteins, and enhances fibroblast proliferation and migration. In the absence of *Fgf13*, the expressions of these proteins are decreased, and fibroblast proliferation and migration are suppressed, compared with those in the TGFβ-stimulated group. Overexpression of FGF13, but not FGF13 mutants defective in microtubule binding and stabilization, rescues the decrease in collagen and α-SMA protein and weakens the proliferation and migration function of the *Fgf13* knockdown group. Furthermore, *Fgf13* knockdown decreases ROCK protein expression via microtubule disruption. Collectively, cardiac *Fgf13* knockdown protects the heart from fibrosis in response to haemodynamic stress by modulating microtubule stabilization and ROCK signaling pathway.

## Introduction

Cardiac fibrosis serves as an integral component of cardiac remodeling, leading to diastolic dysfunction, arrhythmia, and heart failure [1]. It is a devastating condition typified by collagen accumulation in the extracellular matrix [2]. In the course of cardiac fibrosis pathology, cardiac fibroblasts first become activated and subsequently differentiate into myofibroblasts by signaling pathways such as transforming growth factor β (TGFβ) signaling [3], thus results in increased α-smooth muscle actin (α-SMA) expression. Subsequently, myofibroblasts which act as the main mediators of pathological remodeling, migrate to the injured site and produce a large amount of collagen, thereby leading to the pathological process of cardiac structural alterations [ 4– 6]. Therefore, it is of great clinical significance to find effective methods to delay cardiac fibrosis.

Fibroblast growth factor (FGF) 13 is a member of the factor homologous factors (FHFs) family. The four FHFs, a subpopulation of the FGF family, have gained increased interest for their ability to regulate pressure overload heart disease. FGFs interact with the extracellular domains of FGF cell surface receptors (FGFRs) to trigger receptor activation and biological responses. FGF homologous factors (FHF1–FHF4, also referred to as FGF11–FGF14) exhibit substantial sequence homology with FGFs but fail to activate all seven known FGFRs and cannot be secreted [ 7, 8]. FGF13 is the predominant FHF in adult mouse ventricular myocytes. It aids in regulating arrhythmias by controlling the current density of Na ^+^ channels [9] and Ca ^2+^-induced Ca ^2+^ release [10]. Moreover, *Fgf13* knockdown enhances caveolae-mediated cardioprotection during cardiac pressure overload [11]. Additionally, as a microtubule stabilizer, FGF13 regulates neuronal polarization and migration [12], manages inflammatory pain [13], and enhances the resistance of cancer cells to platinum drugs [14]. Although FGF13 is a potentially significant regulator of heart disease, its function in CFs remains unexplored.

A previous study showed that FGF13 is a microtubule stabilizing protein [12]. The stability of tubulin significantly influences fibrosis formation. Itano and his colleagues [15] reported that colchicine attenuates renal fibrosis in a murine unilateral ureteral obstruction model by inhibiting angiotensin II-induced fibroblast migration. Moreover, tubulin proteins are upregulated upon the initial development of endothelium-mesenchymal transition, where they are involved in the inception of mesenchymal behavior by enhancing cell migration [16]. Furthermore, enhanced microtubule stability can activate RhoA and downstream signaling pathways [17], which are essential for fibrosis regulation [18].

Here, we tested the hypothesis that *Fgf13* knockdown can promote fibroblast function by regulating the stability of microtubules and ROCK pathway, and thereby participate in cardiac fibrosis. We observed that *Fgf13*-knockout (KO) mice showed reduced cardiac fibrosis following pressure overload. FGF13 expression was significantly increased in TGFβ-treated fibroblasts. Conditional knockdown of *Fgf13* in CFs inhibited fibroblast activation and function by attenuating microtubule stability. Microtubule depolymerization prevents ROCK pathway activation. This study reveals that FGF13, as a regulator of microtubule stabilization, could regulate the ROCK pathway and fibroblast activation and function, further suggesting that FGF13 may be a new target for cardiac fibrosis treatment.

## Materials and Methods

### Animals

*FGF13* conditional knockout mice were generated through Cre-LoxP-mediated copulation, and the FGF13-LoxP allele mice were mated with myh6-MCM mice. Floxed FGF13 mice (FGF13fl/fl) were conceived in collaboration with Beijing Biocytogen, Co., Ltd. (Beijing, China) by flanking exon 3 of the mouse *Fgf13* gene with two loxP sites. Myh6-mcm mice have an alpha-myosin heavy chain ( *Myh6*) promoter that directs the expression of tamoxifen-induced Cre recombinase. Genomic DNA was isolated from the mouse tail and genotyped as previously described [19]. The animals used in our experiments were adult male C57BL/6J mice aged 8 to 16 weeks. Hemizygous knockout mice (KO) and no floxed wild-type (WT) control mice were intraperitoneally injected with 30 mg/kg tamoxifen (Sigma, St Louis, USA) for 3 days to induce *Fgf13* deletion. Mice recovered from tamoxifen induction for at least one week before the experiment. The mice were randomly allocated into four groups: the control group (WT-sham), the KO group (KO-sham), WT mice that underwent transverse aortic constriction (TAC) surgery (WT-TAC), and KO mice that underwent TAC surgery (KO-TAC). All animal experiments were approved by the Experimental Animal Ethics and Welfare Committee of Hebei Medical University (IACUC-HebMU-2022011, Shijiazhuang, China).

### Generation of pressure overload echocardiography

As previously described [20], pressure overload was established by constricting the transverse aorta. The following is the general process: first, the mice were anesthetized with isoflurane; second, the skin and muscle tissues were cut between the second and third ribs; third, the thymus was separated to expose the aortic arch; finally, a 7-0 silk ligature was attached to a 27-gauge needle for aortic coarctation.

The mice were anesthetized with 1.5% isoflurane and examined by ultrasound at 12 weeks postoperation. Two-dimensional images of the left ventricle were collected at the level of the left ventricular papillary muscle beside the sternum. Left ventricular internal dimension in systole, left ventricular internal dimension in diastole, left ventricular ejection fraction, fractional shortening and other cardiac function parameters were measured [21].

### Histological analysis

The mouse heart tissue was fixed for at least 24 h and then embedded in paraffin wax. Several sections of each heart (3‒5 μm) were prepared, stained with hematoxylin and eosin for histopathology examination or Sirius red and Masson staining for collagen deposition visualization via light microscopy. The collagen volume fraction was determined by calculating the collagen area/total area using ImageJ software.

### Isolation of neonatal rat cardiac fibroblasts

As previously described [20], 1- to 3-day-old Sprague-Dawley rat hearts were harvested. The heart tissue was digested with 0.125% trypsin-EDTA and collagenase II solution for 6 min until the fragments were completely digested. After centrifugation, the cells were resuspended in plates and cultured for 80 min, which allowed cardiac fibroblasts attach to the culture plates. Cultures were grown and passaged in high-glucose medium (Gibco, Carlsbad, USA) supplemented with 10% fetal bovine serum (FBS; Gibco). The third generation (P3) cells were used in the experiments.

### Cell culture

Cells were grown in high-glucose medium under a water-saturated atmosphere of 95% air and 5% CO _2_ at 37°C. The cultured fibroblasts were randomly assigned to six groups and cultured under different conditions. In the control group, the cells were maintained in normal DMEM. In the transforming growth factor β (TGFβ) group, the cells were cultured in culture medium supplemented with TGFβ1 (10 nM; Proteintech, Rosemont, USA) for 48 h. In the TGFβ- *Fgf13* knockdown (TGFβ-kd) group, cells were first cultured in culture medium containing the *Fgf13* knockdown vector before TGFβ1was added. To explore the effect of *Fgf13* knockdown on fibroblasts, two additional groups were added as follows: in the vector group, cells were treated with the *Fgf13* knockdown empty vector. In the vector+TGFβ group, *Fgf13* knockdown empty vector-transfected cells were treated with TGFβ1. Moreover, to explore the relationship between microtubules and fibrosis, two additional groups were added as follows: in the colchicine (COL) group, cells were cultured in culture medium containing COL. In the COL+TGFβ group, COL cells treated with TGFβ1. In addition, to further investigate the molecular mechanisms by which FGF13 regulates fibroblast activation and function via microtubules, four additional groups were added as follows: in the TGFβ-V1 group, cells were first cultured in culture medium containing the *Fgf13* knockdown empty vector (V1) before TGFβ1 was added. TGFβ-kd-V2 group, *Fgf13*-knockdown cells were first cultured in culture medium supplemented with FGF13 overexpression empty vector (V2) before TGFβ1 was added. In the TGFβ-kd-OE group, *Fgf13*-knockdown cells were first cultured in culture medium containing the FGF13 overexpression vector before TGFβ1 was added. In the TGFβ-kd-MUT group, *Fgf13-*knockdown cells were first cultured in culture medium supplemented with the FGF13 mutant vector before TGFβ1 was added.

### Adenovirus vector transfection

Adenovirus vectors were constructed by GeneChem (Shanghai, China) for shRNA-mediated FGF13 downregulation (GV119), FGF13-VY overexpression (GV314), mutant FGF13-VY overexpression (GV314), and their negative controls. Cells were transfected with adenovirus (infection complex [MOI]=10) for 48 h. The vector information can be obtained from the GeneChem website [12].

### Co-immunoprecipitation analysis

According to the instructions for the magnetic beads (Beyotime, Shanghai, China), 500 μL of protein sample and 20 μL of magnetic beads suspension were prepared and then incubated at 4°C overnight. The magnetic beads were removed the next day. The antibodies were added, and the samples were incubated for 2 h before being washed again. Then, 100 μL of buffer was added to the sample, heated at 95°C for 5 min. These samples were subjected to western blot analysis.

### Cell proliferation assay

Cell growth was analyzed by Cell Counting Kit-8 (CCK8) assay. Briefly, cells in each group were collected and seeded into 96-well plate at a density of 5×10 ^3^ cells/well. After 0, 24, 48 and 72 h of incubation, 10 μL of CCK8 reagent (MCE, Monmouth Junction, USA) was added to each well, and the plates were incubated for 2 h. The absorbance of each well was quantified using a microplate reader at a wavelength of 450 nm [22].

### Transwell invasion assay

The migration assay was performed using transwell permeable supports (Corning, Steuben County, USA). A total of 5×10 ^4^ cells from each group were suspended in 200 μL of serum-free medium and seeded into the upper chamber. The lower chambers were filled with 600 μL of medium containing 10% fetal bovine serum. After 24 h of incubation at 37°C and 5% CO _2_, the nonmigratory cells on the top of the membrane were removed using a cotton swab. Membranes containing cells were fixed with 4% paraformaldehyde and stained with a crystal violet solution. The number of cells on the lower membrane was counted at 200× magnification using a microscope [22].

### Western blot analysis

In brief, cardiac tissues and CFs were lysed with RIPA buffer (23HA0090; Seven, Beijing, China) supplemented with protease inhibitors (HY-K0010, 1:100; MCE). The lysates were centrifuged at 8800 *g* for 20 min at 4°C, after which the supernatants were collected. The concentrations of protein in the supernatant were measured using a bicinchoninic acid (BCA) kit (PC0020; SolarBio, Beijing, China). Equal amounts of proteins were subjected to 12% or 10% SDS‒PAGE and electrophoretically transferred to polyvinyl difluoride (PVDF) membranes (IPVH00010; Millipore, Billerica, USA). The membranes were blocked with 5% non-fat milk at room temperature for 2 h, followed by overnight incubation at 4°C with the following primary antibodies diluted in blocking buffer. Antibodies against α-SMA (14395-1-AP, 1:5000; Proteintech), Col1α2 (14695-1-AP, 1:1000; Proteintech), Col3α1 (22734-1-AP, 1:1000; Proteintech), GAPDH (1:10000; Proteintech), FGF13 (1:200; Yenzym, Brisbane, USA), Flag (F1804, 1:1000; Sigma Aldrich, St Louis, USA), Ace-tubulin (T7451, 1:800; Sigma Aldrich), and anti-Detyrosinated tubulin (Detyr-tubulin) (ab254154, 1:1000; Abcam, Cambridge, UK) were used. Subsequently, the immunoblots were incubated with anti-rabbit (D30627-05, 1:10000; LI-COR Biosciences, Lincoln, USA) or anti-mouse (D30613-05, 1:10000; LI-COR Biosciences) IgG-HRP for 2 h at room temperature. The signal intensity was quantified using an Odyssey Infrared Imaging System (LI-COR Biosciences).

### Statistical analysis

The results were analyzed by SPSS 22.0 and GraphPad Prism 8, and the data are presented as the mean±SD. Differences between two groups were analyzed by Student’s *t* test, and differences among multiple groups were analyzed with one-way ANOVA followed by Bonferroni correction for multiple comparisons. *P*<0.05 was considered statistically significant.

## Results

### *Fgf13* knockout hearts maintain cardiac function to cope with chronic pressure overload

**Figure 1:**
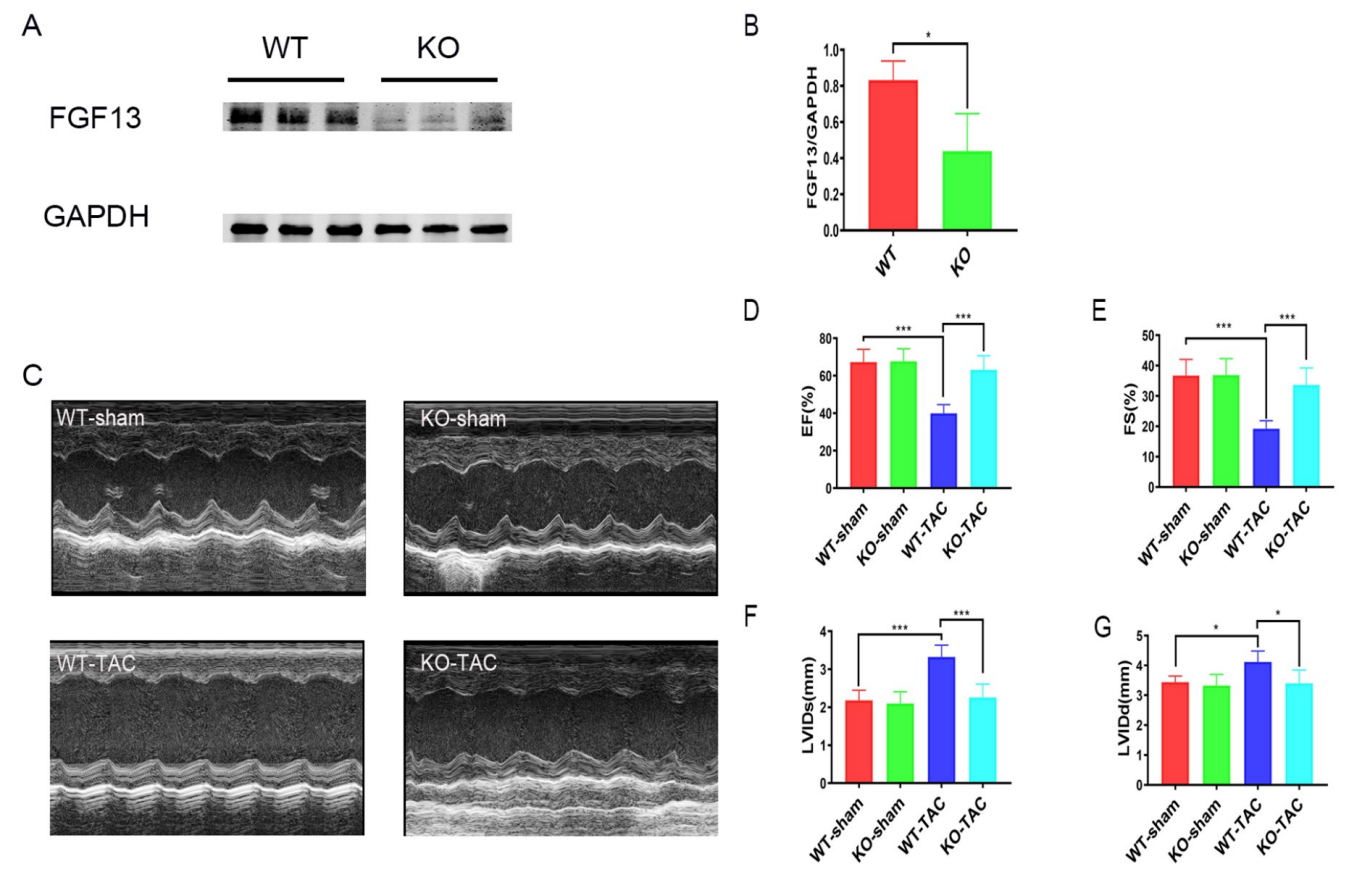
Figure 1 *FGF13*-KO hearts maintain cardiac function under pressure overload (A) Representative images of FGF13 protein expression in heart tissue. (B) Quantification of the FGF13 protein. n=3 in each group. (C) Representative M-type echocardiograms of wild-type control (WT-sham), cardiac knockout (KO) FGF13 mice (KO-sham), wild-type TAC (WT-TAC), and knockout TAC mice (KO-TAC). (D–G) Summarized echocardiographic measurements of (D) ejection fraction (EF), (E) fractional shortening (FS), (F) left ventricular internal dimension in systole (LVIDs), and (G) left ventricular internal dimension in diastole (LVIDd). n=6 for each group. *P<0.05, and ***P<0.001.

To clarify the role of FGF13 in cardiac fibrosis, *Fgf13* conditional KO mice were generated by tamoxifen injection. Measurement of FGF13 protein by western blot analysis demonstrated efficient inducible deletion of FGF13 from heart tissues ( Figure 1A,B). A previous study showed that gender does not contribute to baseline functional differences [11]. Therefore, we used male KO mice in this study. We initially assessed whether *Fgf13* KO contributes to baseline functional differences. Echocardiography revealed no differences in fractional shortening (FS), ejection fraction (EF) as well as left ventricular end systolic and diastolic diameter in *Fgf13*-KO mice compared to controls ( Figure 1C‒G). Consequently, we explored the functional role of *Fgf13* KO in transverse aortic constriction (TAC)-induced pathological cardiac remodeling. Compared with those in control mice, TAC led to severe cardiac dysfunction in C57BL/6 mice by 12 weeks, with a progressive decrease in EF ( Figure 1D), FS ( Figure 1E) and chamber enlargement ( Figure 1F,G). However, ventricular dilation and decreases in the EF at 12 weeks of TAC were significantly diminished when *Fgf13* was selectively deleted from the heart ( Figure 1C‒G). These results suggested that cardio-specific *Fgf13* KO had a protective effect on cardiac function.

### *Fgf13* knockout reduces cardiac fibrosis under chronic pressure overload

**Figure 2:**
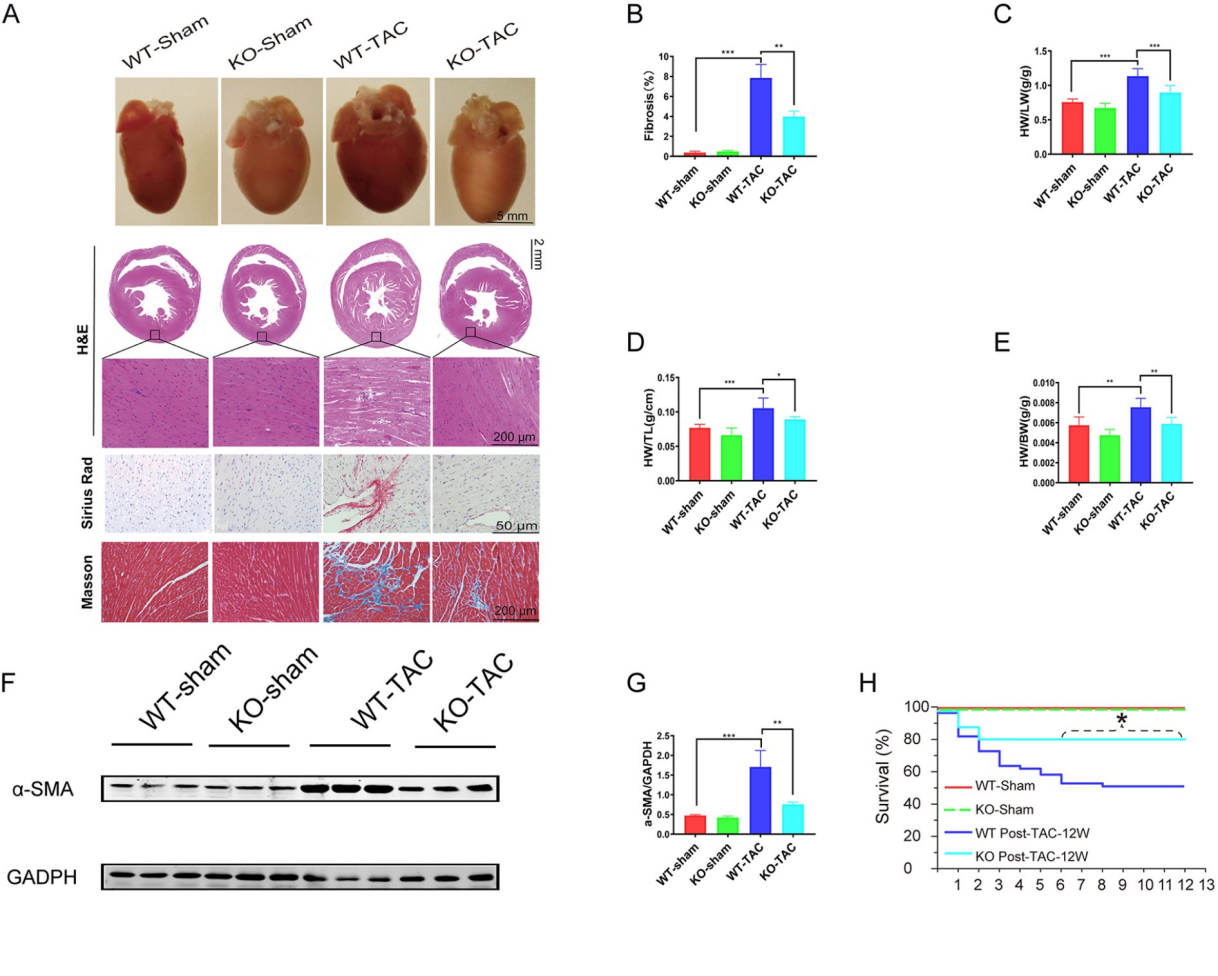
Figure 2 *FGF13* KO reduces cardiac fibrosis under pressure overload (A) Whole mounts of representative hearts from wild-type control mice (WT-sham), cardiac KO FGF13 mice (KO-sham), wild-type TAC (WT-TAC) mice, and knockout TAC mice (KO-TAC). Histological sections were stained with hematoxylin/eosin, Sirius red and Masson’s trichrome. (B) Quantitative results of Masson trichromatic staining. n=3 for each group. (C) Heart-to-lung weight ratio (HW/LW), n=6 in each group. (D) Heart weight normalized to tibial length (HW/TL). n=6 in each group. (E) Heart weight was normalized to body weight (HW/BW). n=6 in each group. (F) Representative images of α-SMA protein expression in heart tissue. (G) Quantification of the α-SMA protein. n=3 in each group. (H) Survival curves after TAC injury in WT or Fgf13 KO mice. n=5 in each group. *P<0.05, **P<0.01, and ***P<0.001.

Diastolic dysfunction is associated with increased stiffness and fibrotic deposition. Therefore, we explored whether *Fgf13* KO could prevent cardiac fibrosis in the face of increased cardiac stress. We used TAC *Fgf13* KO or WT mice and compared them to *Fgf13* KO-sham or WT-sham mice. The results showed that mice subjected to TAC displayed an enlarged heart size, as shown in the whole heart image, HE staining ( Figure 2A), heart weight-to-lung weight ratio ( Figure 2C), heart weight normalized to tibia length ( Figure 2D) and heart weight-to-body weight ratio ( Figure 2E). Furthermore, Sirius red staining and Masson staining revealed significantly more fibrosis in TAC hearts ( Figure 2A,B). Contrarily, the extent of *Fgf13* KO heart mass and fibrosis area were decreased compared to those in TAC hearts. Consistent with these histological findings, *Fgf13* KO attenuated α-SMA accumulation after 12 weeks of TAC, indicating that FGF13 is involved in activation inhibition in cardiac fibroblasts compared with that in the TAC hearts ( Figure 2F,G). Moreover, owing to the protective effects of *Fgf13* KO on the heart, *Fgf13*-KO TAC mice exhibited markedly greater survival than WT TAC mice ( Figure 2H). Overall, these results indicated that mice with *Fgf13* conditional KO in the heart exhibited significantly reduced cardiac fibrosis in response to pressure overload.

### *Fgf13* knockdown relieves TGFβ1-induced fibroblast activation and function in neonatal rat cardiac fibroblasts

**Figure 3:**
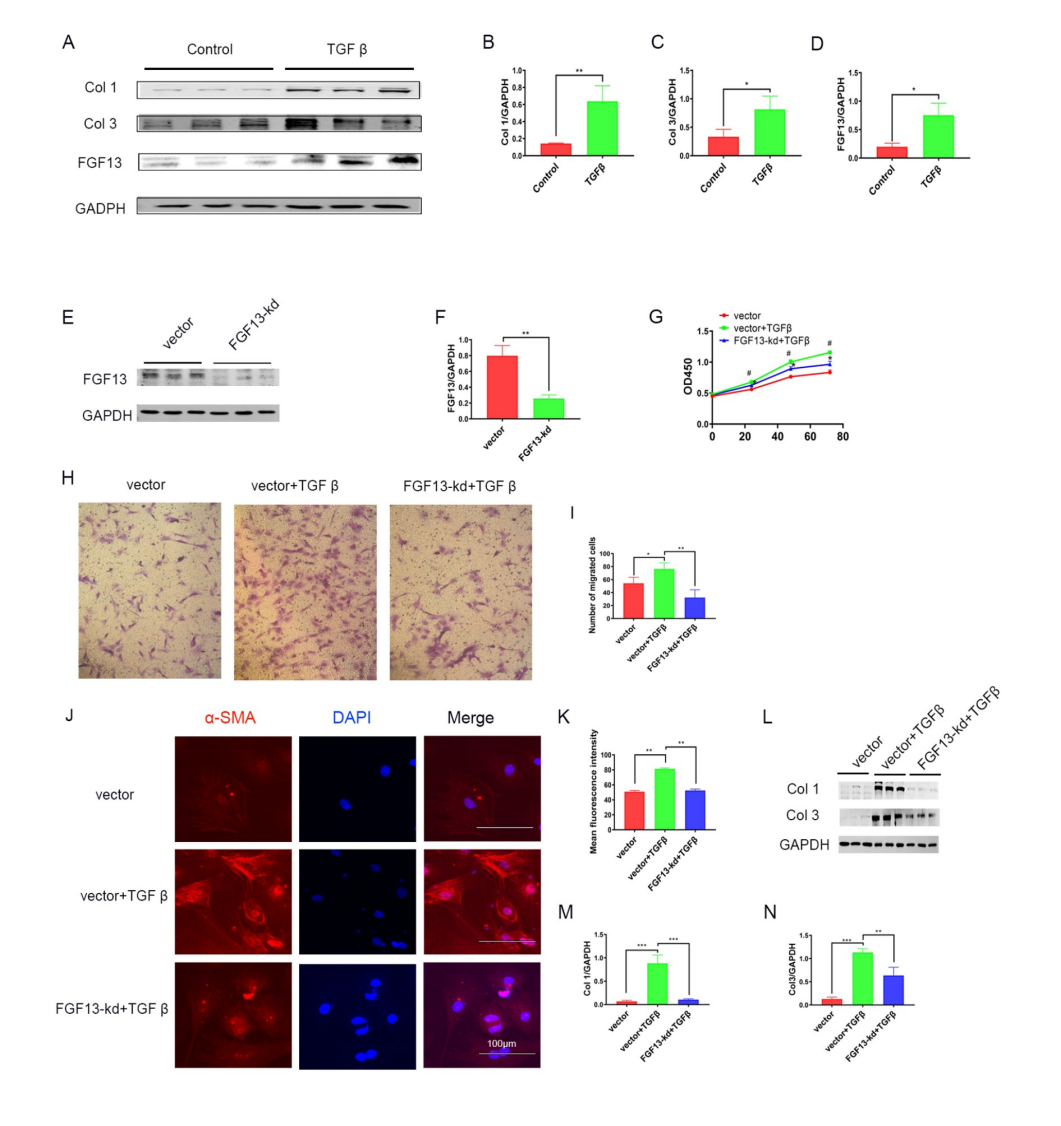
Figure 3 *FGF13* knockdown inhibits fibroblasts activation and function in TGFβ1 pre-stimulated CFs (A) Representative images of collagen and FGF13 expression in fibroblasts. Control, cells treated with PBS; TGFβ, cells treated with TGFβ1. (B) Quantitative analysis of type I collagen. n=3 in each group. (C) Quantitative analysis of type III collagen. n=3 in each group. (D) Quantitative analysis of the FGF13 protein. n=3 in each group. (E) Representative images of FGF13 expression in fibroblasts. Vector, cells treated with the FGF13 knockdown empty vector; FGF13-kd, cells treated with the FGF13 knockdown virus. (F) Quantitative analysis of the FGF13 protein. n=3 in each group. (G) The proliferation of CFs was analyzed by CCK8-Kit assay. Vector, cells treated with the FGF13 knockdown empty vector; vector+TGFβ, FGF13 knockdown empty vector cells treated with TGFβ1; FGF13-kd+TGFβ, FGF13-knockdown cells treated with TGFβ1. n=3 in each group. #P<0.05 compared with the vector group; *P<0.05 compared with the vector+TGFβ group. (H,I) Representative micrographs and quantification of migrating cells after crystal violet staining. n=3 in each group. Magnification fold: 100×. (J,K) Immunofluorescence staining and quantification of α-SMA expression. n=3 in each group. Scale bar: 100 μm. (L) Representative images of collagen expression in fibroblasts. (M,N) Protein quantification of collagen type I (M) and collagen type III (N). n=3 for each group. *P<0.05, **P<0.01, and ***P<0.001.

To determine whether *Fgf13* knockdown directly affects CFs to protect against cardiac fibrosis, CFs were isolated and treated with TGFβ1, followed by cultivation with *Fgf13* knockdown virus. We first examined the alterations in collagen and FGF13 expressions in a TGFβ1-induced fibrosis model *in vitro*. Using western blot analysis, we found that TGFβ1-treated CFs showed significantly increased collagen type I and III protein expressions ( Figure 3A‒C). In addition, analysis of extracts of TGFβ1-treated CFs showed higher FGF13 protein abundance compared with the control group ( Figure 3A,D). Then, we examined fibroblast activation and function in TGFβ1-prestimulated CFs. The results showed that TGFβ1-treated media induced proliferation ( Figure 3G), migration ( Figure 3H,I) and myofibroblast differentiation (detected as α-SMA, Figure 3J,K). Subsequently, we investigated the effects of *Fgf13* knockdown on CF activation and function. Through western blot analysis, we observed lower FGF13 expression upon incubation with *Fgf13* knockdown virus ( Figure 3E,F). Interestingly, in TGFβ1-stimulated cells, *FGF13* knockdown decreased both cell migration ( Figure 3H,I) and proliferation ( Figure 3G) and reduced collagen levels ( Figure 3L‒N) in CFs. Moreover, *Fgf13* knockdown markedly reduced CF activation, as reflected by decreased α-SMA ( Figure 3J,K). These results indicate that *Fgf13* knockdown inhibits collagen protein production and fibroblast activation and function.

### FGF13 promotes microtubule stabilization in cardiac fibrosis

**Figure 4:**
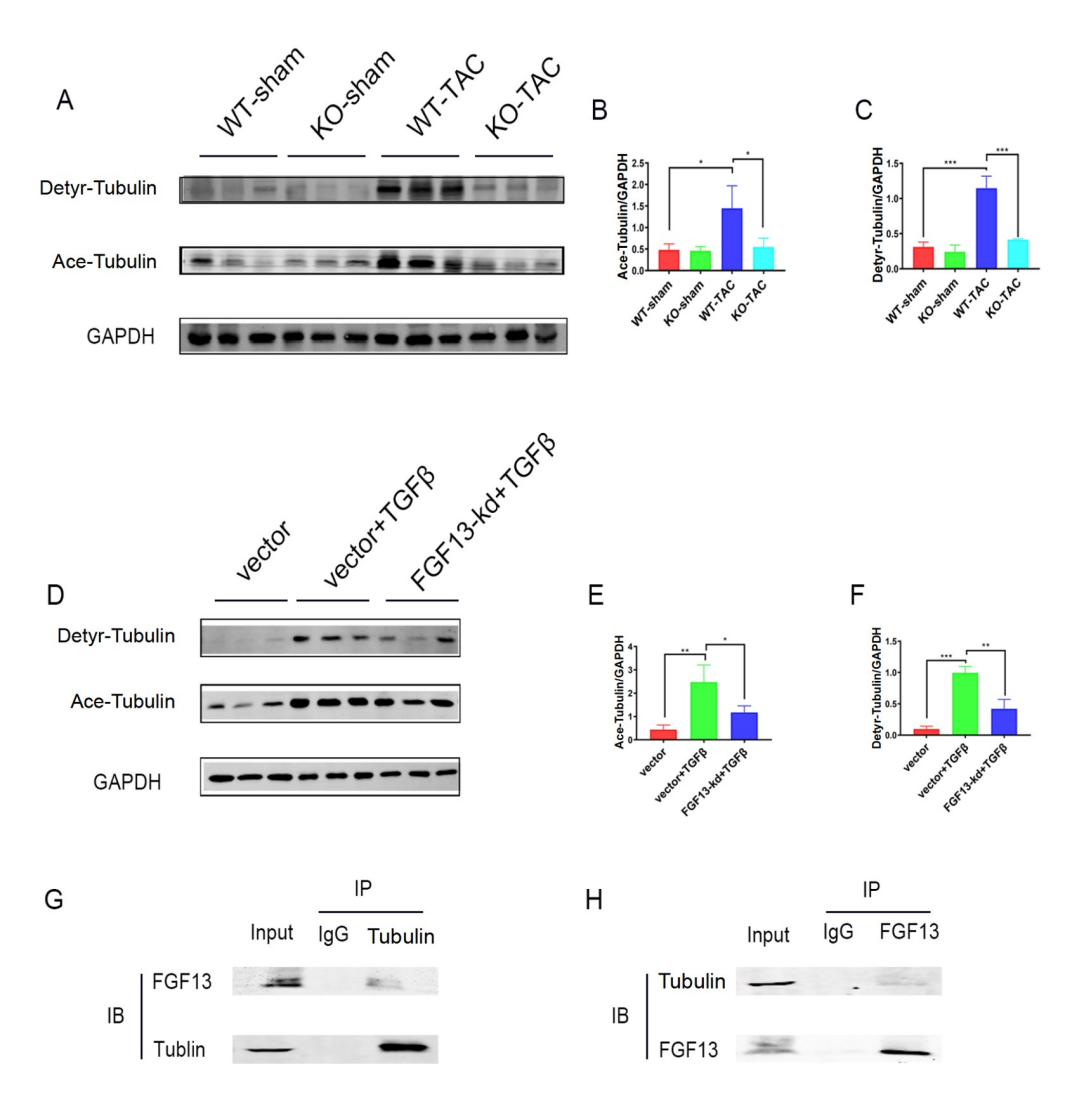
Figure 4 FGF13 promotes microtubule stabilization in cardiac fibrosis (A‒C) Wild-type control (WT-sham), cardiac knockout FGF13 mice (KO-sham), wild-type TAC (WT-TAC), and knockout TAC mice (KO+TAC). Expression and quantification of the Ace-tubulin and Detyr-tubulin proteins in the heart. (B) Quantification of Ace-tubulin. n=3 in each group. (C) Quantification of Detyr-tubulin. n=3 in each group. (D‒F) Expression of the Ace-tubulin and Detyr-tubulin proteins in fibroblasts. Vector, cells treated with the FGF13 knockdown empty vector; vector+TGFβ, FGF13 knockdown empty vector cells treated with TGFβ1; FGF13-kd+TGFβ, FGF13-knockdown cells treated with TGFβ1. (E) Quantification of Ace-tubulin. n=3 in each group. (F) Quantification of Detyr-tubulin. n=3 in each group. (G,H) The ability of FGF13 to bind to tubulin was confirmed by co-IP. *P<0.05, **P<0.01, and ***P<0.001.

Microtubules affect cell proliferation and migration and are involved in the occurrence and development of heart failure. In our study, the expressions of acetylated tubulins and detyrosinated tubulins were elevated in the heart following TAC surgery ( Figure 4A‒C). Similarly, the levels of these two proteins significantly increased in TGFβ1-treated CFs ( Figure 4D‒F). Previous studies reported that FGF13 induced microtubule polymerization and stabilization [12]. Therefore, we subsequently investigated whether FGF13 has a microtubule stabilization effect on cardiac fibrosis. We noted that acetylated and detyrosinated tubulin levels decreased in *Fgf13*-KO hearts induced by pressure overload compared to those in the TAC group ( Figure 4A‒C). Furthermore, when exposed to TGFβ1, CFs with *Fgf13* knockdown showed lower acetylated and detyrosinated tubulin levels compared with those in the TGFβ1 group ( Figure 4D‒F). Finally, we performed co-IP experiment, and the interaction between FGF13 and microtubules was detected in CFs ( Figure 4G,H). Overall, these results indicated that FGF13 promotes microtubule stabilization in cardiac fibrosis.

### Effects of microtubule depolymerization on CF activation and function

**Figure 5:**
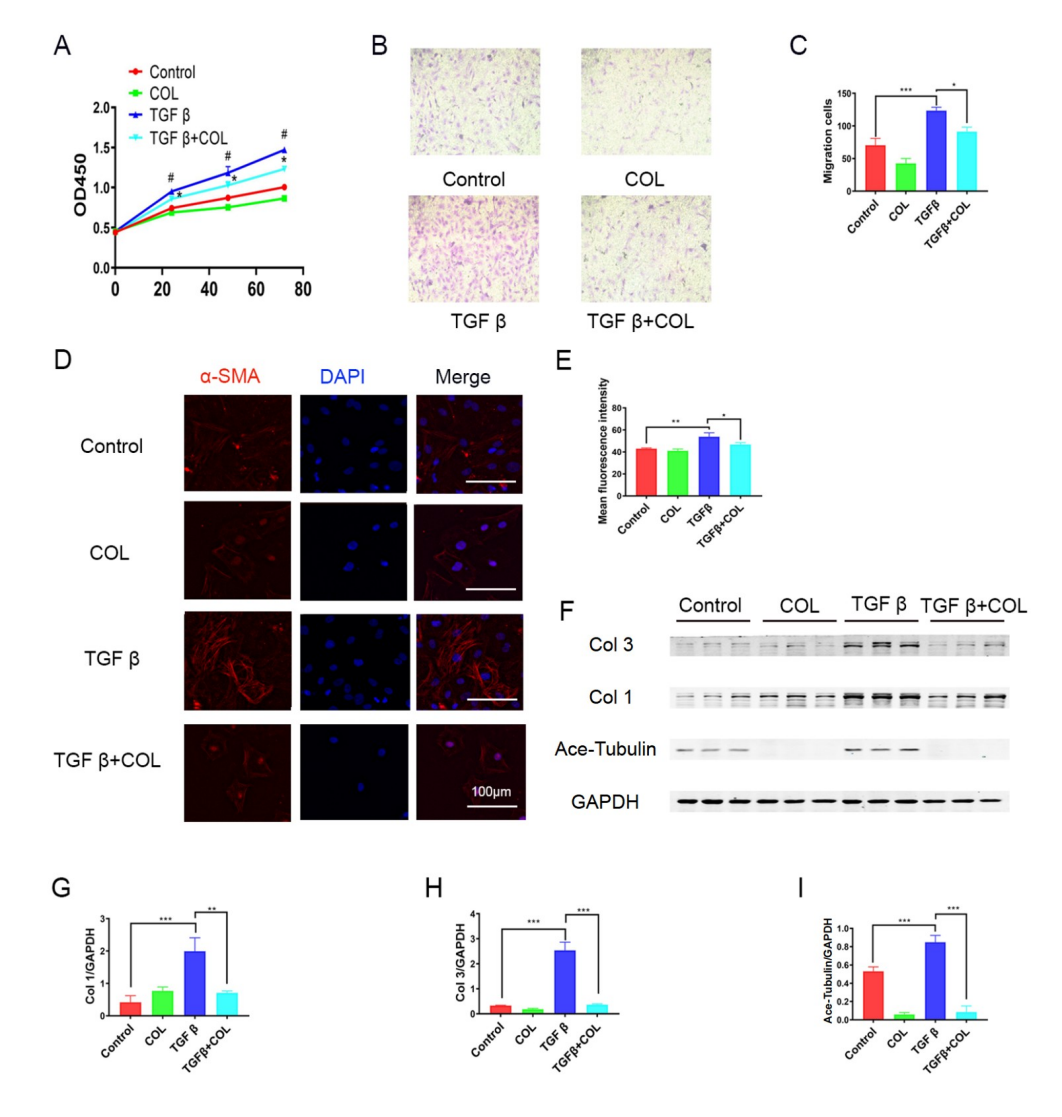
Figure 5 Microtubule depolymerization inhibits fibroblasts activation and function during the fibrotic response (A) Control, cells treated with PBS; colchicine (COL), cells treated with COL; TGFβ, cells treated with TGFβ1; COL+TGFβ, COL cells treated with TGFβ1. The proliferation of CFs was analyzed by CCK8-Kit assay. n=3 in each group. #P<0.05 compared with the control group; *P<0.05 compared with the TGFβ group. (B,C) Representative images and quantification of migrating cells after crystal violet staining. n=3 for each group. Magnification fold: 100×. (D,E) Immunofluorescence staining and quantification of α-actin. n=3 in each group. Scale bar: 100 μm. (F) Representative images of collagen expression in fibroblasts; (G-I) Quantitative results of protein; (G) collagen type I. n=3 in each group. (H) Collagen type III. n=3 in each group. (I) Ace-Tubulin. n=3 in each group. *P<0.05, **P<0.01, and ***P<0.001.

Having revealed the regulatory effects of FGF13 on microtubules in the heart, we subsequently investigated whether the changes in CF activation and function are caused by microtubule depolymerization. To explore the relationship between microtubules and fibrosis, we utilized the microtubule depolymerizing agent colchicine as a surrogate for *Fgf13* knockdown. Notably, it was found that the proliferation and migration of CFs were inhibited by colchicine during the fibrotic response ( Figure 5A‒C). Furthermore, in TGFβ1-treated CFs, colchicine stimulation resulted in a significant decrease in α-SMA expression ( Figure 5D,E) and a downregulation of extracellular matrix-related proteins, including type I and III collagen ( Figure 5F‒I), indicating that fibroblast activation is inhibited by colchicine. In general, the abovementioned data suggested that microtubule depolymerization inhibits CF activation and function in the fibrotic response.

### FGF13 regulates fibroblast activation and function via microtubules

**Figure 6:**
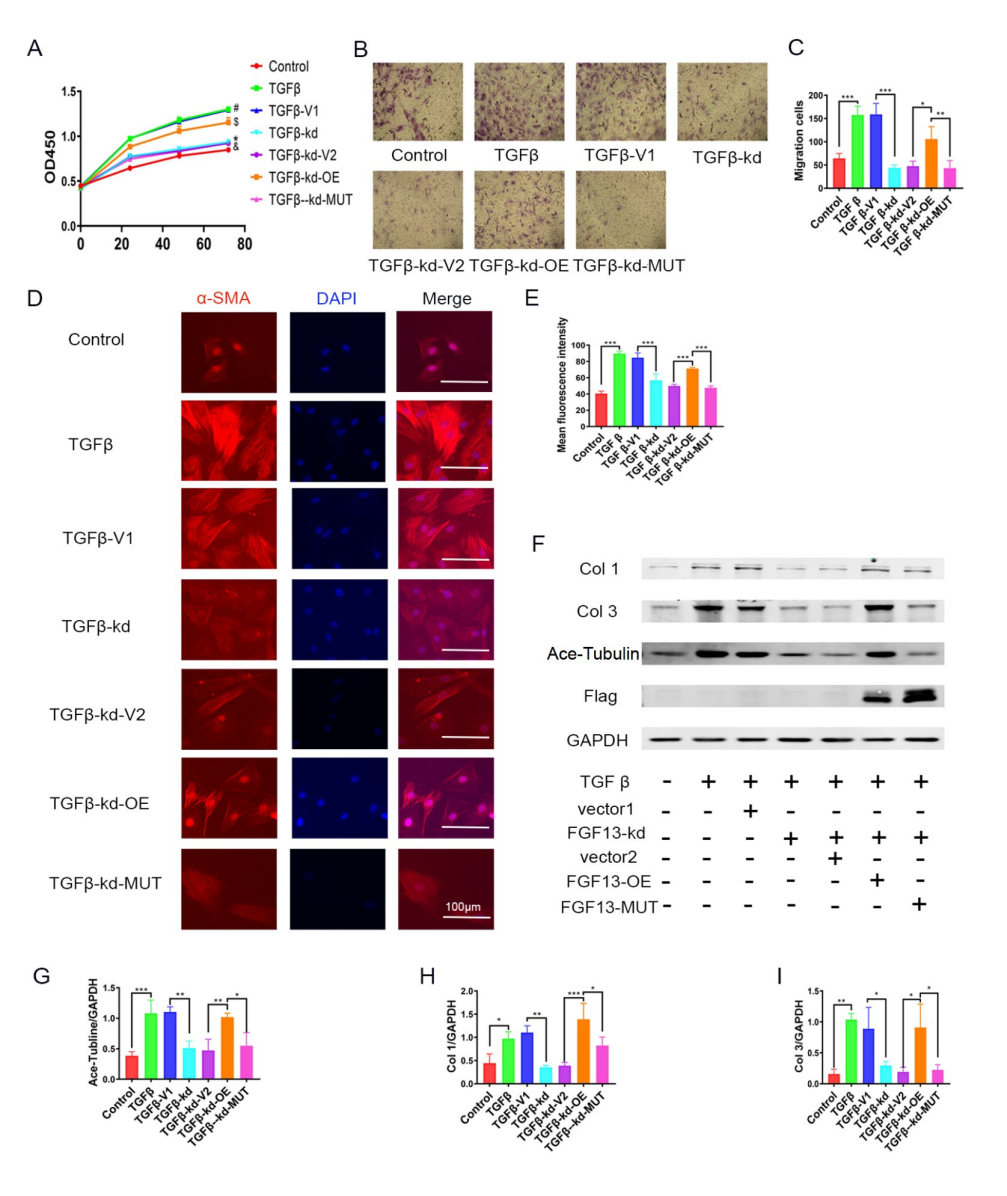
Figure 6 FGF13 regulates fibroblast activation and function through microtubules (A) Control, cells treated with PBS; TGFβ, cells treated with TGFβ1; TGFβ-V1, Fgf13 knockdown empty vector (V1) cells treated with TGFβ1; TGFβ-kd, Fgf13-knockdown cells treated with TGFβ1; TGFβ-kd-V2, Fgf13-knockdown cells treated with FGF13 overexpression and mutant empty vector (V2) and TGFβ1; TGFβ-kd-OE, Fgf13-knockdown cells treated with FGF13 overexpression virus and TGFβ1; TGFβ-kd-MUT, Fgf13-knockdown cells treated with FGF13 mutant virus and TGFβ1. The proliferation of CFs was analyzed by CCK8-Kit assay. n=3 for each group. #P<0.05 compared with the control group; *P<0.05 compared with the TGFβ-V1 group; $P<0.05 compared with the TGFβ-kd-V1 group; &P<0.05 compared with the TGFβ-kd-OE group. (B,C) Representative images and quantification of migrating cells after crystal violet staining. n=3 for each group. Magnification fold: 100×. (D,E) Immunofluorescence staining and quantification of α-SMA. n=3 in each group. Scale bar: 100 μm. (F‒I) Representative images and quantitative results of collagen type I (G), collagen type III (H), and Ace-tubulin (I). n=3 in each group. *P<0.05, **P<0.01, and ***P<0.001.

To test this hypothesis, rescue experiments were conducted. First, we infected CFs with a Flag-tagged *Fgf13* and *Fgf13* mutant adenovirus lacking a microtubule binding domain. Next, we established a fibrosis cell model with TGFβ1 *in vitro*. CCK8 assays revealed a protective effect on CF proliferation in the *Fgf13* knockdown group compared with that in the TGFβ1 group. Wild-type *Fgf13* overexpression rescued CF proliferation inhibition, whereas no change in CF quantity was observed when the *Fgf13* mutant was used to infect the *Fgf13*-deficient CFs ( Figure 6A). Furthermore, transwell assay was used to confirm migration, and the results suggested that CF migration was weakened following *Fgf13* gene knockdown. However, *Fgf13* overexpression, not FGF13 mutation, reversed the decrease in CF migration ( Figure 6B,C). We next focused on the expressions of proteins. In response to TGFβ1 stimulation, *Fgf13* overexpression eliminated the protective effects of *FGF13* knockdown on CFs, as shown by the increased expression levels of α-SMA ( Figure 6D,E) and collagen types I and III ( Figure 6F‒I) in the *Fgf13* overexpression group but no changes in the *Fgf13* mutant group compared with the *Fgf13* knockdown group. These data indicated that FGF13 could regulate microtubule-mediated fibroblast activation and function.

### The interaction between FGF13 and microtubules regulates cardiac fibrosis through the ROCK pathway

**Figure 7:**
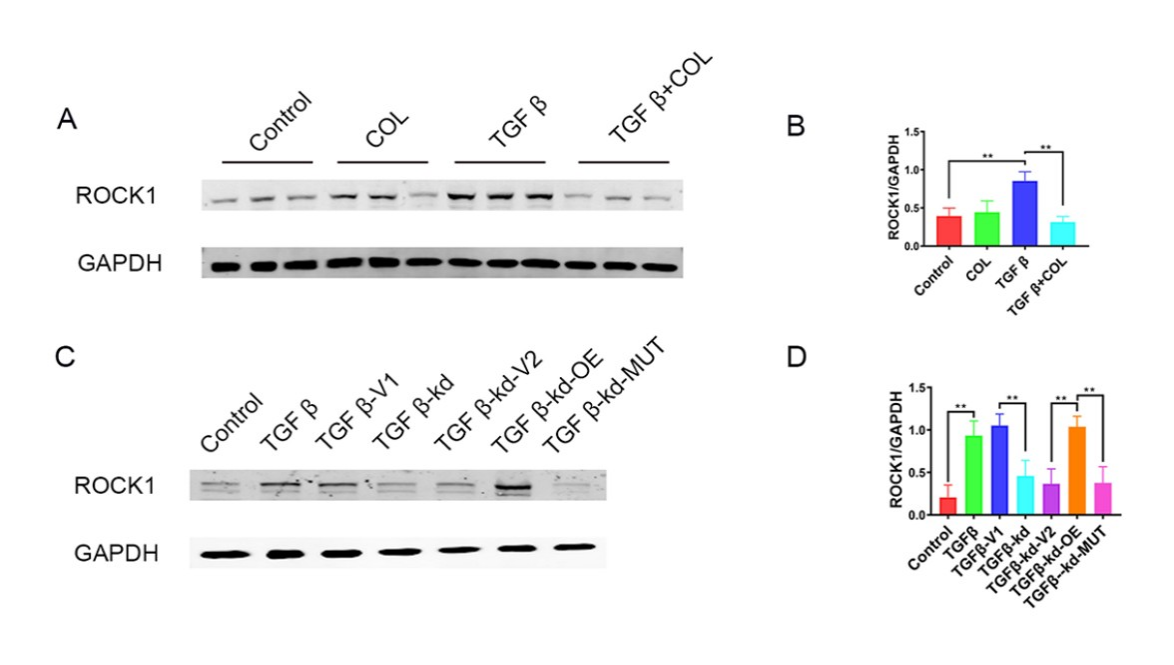
Figure 7 FGF13 regulates the ROCK pathway through microtubules (A) The expression of ROCK protein in fibroblasts. Control, cells treated with PBS; COL, cells treated with COL; TGFβ, cells treated with TGFβ1; COL+TGFβ, COL cells treated with TGFβ1. (B) Quantitative results of ROCK. n=3 in each group. (C) Representative images of ROCK protein expression. Control, cells treated with PBS; TGFβ, cells treated with TGFβ1; TGFβ-V1, FGF13 knockdown empty vector (V1) cells treated with TGFβ1; TGFβ-kd, Fgf13-knockdown cells treated with TGFβ1; TGFβ-kd-V2, FGF13-knockdown cells treated with FGF13 overexpression and mutant empty vector (V2) and TGFβ1; TGFβ-kd-OE, Fgf13-knockdown cells treated with FGF13 overexpression virus and TGFβ1; TGFβ-kd-MUT, Fgf13-knockdown cells treated with FGF13 mutant virus and TGFβ1. (D) Quantitative results. n=3 in each group. **P<0.01.

The RhoA/ROCK pathway, a mechanosensitive signaling pathway in cardiac fibrosis [6], is regulated by microtubules [17]. We therefore investigated whether the ROCK pathway participates in FGF13 regulation in cardiac fibrosis. Western blot analysis revealed upregulated ROCK1 protein level in TGFβ1-treated CFs ( Figure 7A,B). Conversely, colchicine reduced the amount of ROCK1 stimulated by TGFβ1 ( Figure 7A,B). Ultimately, to explore the relationships among ROCK, FGF13 and microtubules, we performed rescue experiments. The results showed that *Fgf13* overexpression rescued the decrease in ROCK1 expression, whereas no change was observed when the FGF13 mutant was used to infect the *Fgf13*-deficient CFs ( Figure 6A). Taken together, the abovementioned results indicated that *Fgf13* knockdown inhibits the upregulation of ROCK1 protein expression by depolymerizing microtubules.

## Discussion

Cardiac fibrosis can progress into debilitating conditions that dramatically affect patients’ quality of life. The FGF family has gained increased interest owing to its role in fibrosis regulation [ 23, 24]. However, FGF13, as an intracellular protein lacking the signal peptide for secretion and function, has remained unexplored in cardiac fibrosis. In this study, we suspected that *Fgf13* conditionally KO could modulate cardiac fibrosis induced by stress overload. Moreover, *Fgf13* knockdown relieved TGFβ1-induced fibroblast activation and function in neonatal rat cardiac fibroblasts. Additionally, *Fgf13* overexpression, but not FGF13 mutation, reversed the decreases in fibroblast activation, function, and collagen as well as ROCK protein production induced by *Fgf13* knockdown. This study indicated that FGF13 can influence fibroblast function and decrease ROCK pathway activation by stabilizing microtubules, thereby regulating cardiac fibrosis.

It has been reported that left ventricular pressure overload, leading to progressive interstitial and perivascular fibrosis, is associated with markedly reduced myocardial compliance and increased myocardial stiffness [13]. Consistently, our studies revealed that TAC mice showed a progressive decline in diastolic function and increased fibrosis. FGF13 has been reported to be the most abundant FHF in the heart [9], which regulates heart hypertrophy signaling pathways, including the caveolae, and NF-κB pathways, so as to change cardiac hypertrophy [ 11, 25, 26]. Similarly, our data revealed that *Fgf13*-KO mice exhibited preserved contractile function and decreased fibrosis at 12 weeks post TAC, indicating that *Fgf13* conditional KO in the heart displayed significantly improved cardiac function and reduced fibrosis in response to pressure overload. However, at baseline, KO mice showed no significant differences in cardiac function, fibrosis or microtubule stabilization, when compared with control mice. This observation may be attributed to the fact that the cardiac fibrosis response is coordinated by a complex fibrosis network that comprises several signaling pathways [9].

Microtubules play a significant role in intracellular transport [27]. Studies have shown that colchicine reduces inflammatory factor transport by depolymerizing microtubules and is used in the treatment of cardiovascular diseases such as atrial fibrillation and fibrosis [ 28, 29]. In our study, colchicine maintained the activation, function and protein production of CFs in the fibrotic response through depolymerizing microtubules, which is similar to the effect of *Fgf13* knockdown on TGFβ-treated CFs. FGF13 regulates microtubule stability and interacts with microtubules in diverse cells, including DRG neurons, spinal cord neurons, and the A549 cell line, thereby affecting disease occurrence [ 12, 14, 30]. However, this interplay has yet to be elucidated in fibroblasts. Therefore, we performed co-IP and rescue experiments and noted that FGF13 could promote fibroblast activation and function by interacting with and stabilizing microtubules in CFs, which provided novel mechanistic insights into how FGF13 regulates cardiac fibrosis under pressure overload. However, additional mechanisms involved in fibroblast function regulation by FGF13 remain to be discovered. A previous study showed that fibrotic signals require p38 MAPK for myofibroblast differentiation [31]. Moreover, FHFs have been reported to aid in the recruitment of p38 to IB2 and may serve as kinase substrates [32]. Therefore, whether FGF13 can also regulate p38 MAPK-mediated fibroblast activation and function may be interesting.

Previous studies reported that RhoA/ROCK signaling activation (via Smad-independent pathways) mediates some of the effects of TGFβ on cardiac fibroblasts [ 5, 33]. Moreover, paclitaxel injection obviously activated the RhoA/ROCK signaling pathway in paclitaxel-induced hypersensitivity [17]. To examine the relationships among ROCK, microtubules and FGF13, we quantified ROCK1 expression. The results showed that *Fgf13* knockdown inhibited ROCK1 protein upregulation by depolymerizing microtubules, indicating that ROCK is involved in FGF13 regulation in cardiac fibrosis via microtubules. Nevertheless, it has been demonstrated that RhoA and its effector ROCK are known for promoting stress fiber formation and actomyosin contractility through pMLC [34]. Microtubule acetylation promotes GEF-H1 release from microtubules, which increases RhoA activity [35]. Therefore, investigating the detailed mechanism by which FGF13 regulates the RhoA/ROCK signaling pathway may be interesting.

Although these discoveries elucidate the discernible antifibrotic function and precise mechanisms of FGF13 in pressure overload-induced cardiac fibrosis, limitations exist. It is easy to injure large blood vessels and pleura during TAC surgery, thereby resulting in hypovolemic shock and pneumothorax. Therefore, the role of FGF13 in other fibrosis models should be explored.

In conclusion, this study showed that FGF13 stabilizes microtubules and upregulates ROCK1 to modulate fibroblast activation and function, thereby modulating cardiac fibrosis. This study revealed the molecular mechanism underlying the contribution of FGF13 to cardiac fibrosis and suggested that FGF13 represents a novel therapeutic target for cardiac fibrosis treatment.
